# Regional Variation in Pregnancy Outcomes amongst Women in Inflammatory Bowel Disease: A Population-Based Cohort Study

**DOI:** 10.1155/2021/3037128

**Published:** 2021-11-29

**Authors:** Parul Tandon, Christina Diong, Rachel Y. Chong, Geoffrey C. Nguyen

**Affiliations:** ^1^Mount Sinai Centre for Inflammatory Bowel Disease, Division of Gastroenterology and Hepatology, University of Toronto, Toronto, Ontario, Canada; ^2^Institute for Clinical Evaluative Sciences, Toronto, ON, Canada; ^3^Department of Medicine, Lakeridge Health, Oshawa, ON, Canada; ^4^Institute of Health Policy, Management and Evaluation, University of Toronto, Toronto, ON, Canada

## Abstract

**Background:**

Women with inflammatory bowel disease (IBD) are at risk of certain pregnancy outcomes such as preterm delivery, infants small for gestational age (SGA), and Cesarean delivery. Whether regional variation in these outcomes exists remains unknown. We aimed to assess the geographical variation in these pregnancy outcomes in women with IBD.

**Methods:**

All pregnancies in women with and without IBD (2002-2013) were identified using Ontario health administrative datasets. Geographical variation in preterm delivery, infants SGA, and Cesarean delivery was assessed using age-adjusted odds ratios (aOR) with 95% confidence intervals (CI) comparing women with and without IBD, stratified by Ontario's 14 health-service regions, known as Local Health Integration Networks (LHINs).

**Results:**

1621 women with IBD (2466 pregnancies) and 855,425 women without IBD (1,280,493 pregnancies) were included. Women with IBD were more likely to have preterm delivery (aOR 1.56, 95% CI, 1.35–1.79), infants SGA (aOR 1.52, 95% CI, 1.23–1.88), and Cesarean section (aOR 1.34, 95% CI, 1.22–1.49). Significant geographical variation in these outcomes was detected, with the highest rates observed in the most northern rural areas (aOR for preterm delivery 2.78 (95% CI, 1.03–7.46), aOR for SGA 5.66 (95% CI, 1.67–19.14), and aOR for Cesarean delivery 2.48 (95% CI, 1.11–5.55)). There were no differences in these outcomes in women with and without IBD in more central urban LHINs.

**Conclusion:**

Significant regional variation was detected in rates of adverse pregnancy outcomes and Cesarean delivery in women with IBD. Further study is required to determine specific reasons for this variation.

## 1. Introduction

Inflammatory bowel disease (IBD) is a chronic inflammatory condition that can affect individuals in the height of their reproductive years [1]. The peak onset of IBD occurs between the second and third decade of life [1], with over half of all patients diagnosed before the age of 35 [2]. Furthermore, over a quarter of women with IBD become pregnant after their IBD diagnosis [3].

Recent studies have demonstrated poor pregnancy outcomes in women with IBD compared to the general population [3]. In particular, the risk of infants born preterm (delivery <37 weeks' gestation) or small for gestational age (SGA) is more than twofold increased in women with active IBD [4]. These adverse outcomes may significantly increase the risk of infant mortality or lead to long-term childhood sequelae such as neurodevelopmental disorders [5].

Similarly, women with IBD are also at a twofold higher risk of undergoing Cesarean delivery than those without IBD [6]. Though indications for Cesarean delivery include active perianal disease or prior ileal-anal anastomosis [7], most women with IBD undergo Cesarean delivery due to fetal indications such as failure of descent, breech presentation, and fetal heart-rate deceleration [8].

However, it remains unknown whether geographical variation exists in the risk of these obstetrical outcomes, particularly in Canada, where the prevalence of IBD is amongst the highest in the world [9]. In Ontario, the largest province by population in Canada, access to specialty care remains a challenge, with wait times exceeding recommended benchmarks [10] and increased hospitalizations and fewer gastroenterology visits in low population density areas [11]. Furthermore, in women without IBD, the rural residence has been associated with significant maternal and neonatal morbidity [12]. Whether this disparity in access to care translates into poor adverse pregnancy outcomes in women with IBD remains to be determined. As such, this study aimed to determine regional variation in rates of preterm delivery, SGA, and Cesarean delivery in women with IBD.

## 2. Methods

### 2.1. Study Design and Data Sources

This was a retrospective, population-based cohort study using province-wide linked administrative data to assess pregnancy outcomes among women in Ontario between April 1, 2002, and March 31, 2012. This project was conducted under section 45 of Ontario's Personal Health Information Protection Act and approved by the Institute for Clinical Evaluative Sciences' (IC/ES) Privacy and Compliance Office.

We used administrative datasets from Ontario that enable access to healthcare records for more than 99% of Ontario's 13.4 million residents who qualify for universal single-payer healthcare [13]. These datasets are stored and analyzed at a health data repository called ICES [14]. Each resident of Ontario is provided a unique identification number based on encrypted health card data, which is then used to link health data across various datasets. The Ontario Crohn's and Colitis Cohort (OCCC) is an inception cohort of IBD patients living in Ontario since 1991, derived from other healthcare administrative databases using validated algorithms with sensitivity and specificity of 76.8% and 96.2%, respectively [15]. The Canadian Institute for Health Information Discharge Abstract Database (CIHI-DAD) contains records for all hospitalizations in Ontario, including those for obstetrical deliveries. The MOMBABY database is an ICES-derived registry of mothers who delivered in Ontario and is linked to birth records of their offspring [16]. The Registered Person's Database (RPDB) includes birth data and postal code of residence and records deaths and migrations out of the province. These datasets were linked using unique encoded identifiers and analyzed at ICES.

### 2.2. Study Population

The source population included all women aged 18 years or more who had given birth in Ontario between April 1, 2002, and March 31, 2012. From this population, two cohorts were created. The IBD cohort included women who delivered in Ontario and were diagnosed after April 1, 1999, as identified by linkage to the OCCC. Within this cohort, IBD cases were further stratified by subtype, ulcerative colitis (UC) or Crohn's disease (CD), based on the assigned diagnostic codes at the last five of nine outpatient physician visits, which is accurate in 91.1% of cases [15]. The non-IBD control cohort included all other women from the source population who were not registered in the OCCC and had no diagnosis codes for IBD in OHIP or CIHI-DAD.

### 2.3. Outcomes

The main obstetrical outcomes assessed included the rates of preterm delivery, infants born SGA, and Cesarean delivery in women with and without IBD. Among the IBD cohort, only births that occurred after IBD was diagnosed were considered in the analysis. Preterm delivery was defined as birth at a gestational age of <37 weeks based on the infant's gestational age at birth recorded in MOMBABY and CIHI-DAD. SGA was defined as below the 3^rd^ percentile for birth weight for a given gestational age. Cesarean delivery was ascertained by linkage to CIHI-DAD and CCI procedural codes (5. MD.60).

We further assessed geographic variation in these obstetrical outcomes using Ontario's Local Health Integration Networks (LHINs) as the unit of analysis. These LHINs correspond to 14 geographically defined areas responsible for funding, planning, managing, and delivering hospital and community-based health services to all residents of Ontario.

### 2.4. Statistical Analysis

Mean neighbourhood income quintiles were calculated for women with and without IBD and reported from Q1 (lowest income quintile) to Q5 (highest income quintile). Mean age with standard deviation (SD) was also reported for the two groups. The rates of preterm delivery, SGA, and Cesarean delivery were calculated by dividing the number of total events of each outcome by the total number of births. The unit of analysis was birth, and as such women may have contributed more than one birth to the overall analysis. The Cesarean delivery rate was further stratified by IBD diagnosis (UC vs. CD) as the presence of active perianal CD is an indication for Cesarean delivery [7]. Categorical variables were analyzed using chi-square statistics, and age-adjusted odds ratios (aOR) with 95% confidence intervals (CI) were calculated to estimate the association between an IBD diagnosis and preterm delivery, SGA, and Cesarean delivery. Finally, rates of preterm delivery, SGA, and Cesarean delivery were further stratified by the 14 LHINs in Ontario, and aORs were calculated for each geographical region. All statistical analysis was conducted using SAS v.9.3. *p* values < 0.05 were considered statistically significant.

## 3. Results

A total of 1621 women with IBD were included (2466 pregnancies) and compared to 855,425 women without IBD (1,280,493 pregnancies). The mean age of included patients was 28.9 (SD 4.5) for women with IBD and 28.8 (SD 5.4) for those without IBD. The mean neighbourhood income quintiles were similar between the two groups (Table 1). Overall, compared to those without IBD, women with IBD had higher odds of delivering preterm (11.9% vs. 8.0%, aOR 1.56, 95% CI, 1.35–1.79), infants born SGA (4.7% vs. 3.0%, aOR 1.52, 95% CI, 1.23–1.88), and Cesarean delivery (37.2% vs. 32.6%, aOR 1.34, 95% CI, 1.22–1.49) (Table 2).

### 3.1. Rates of Preterm Birth Stratified by LHIN

**Figure 1:**
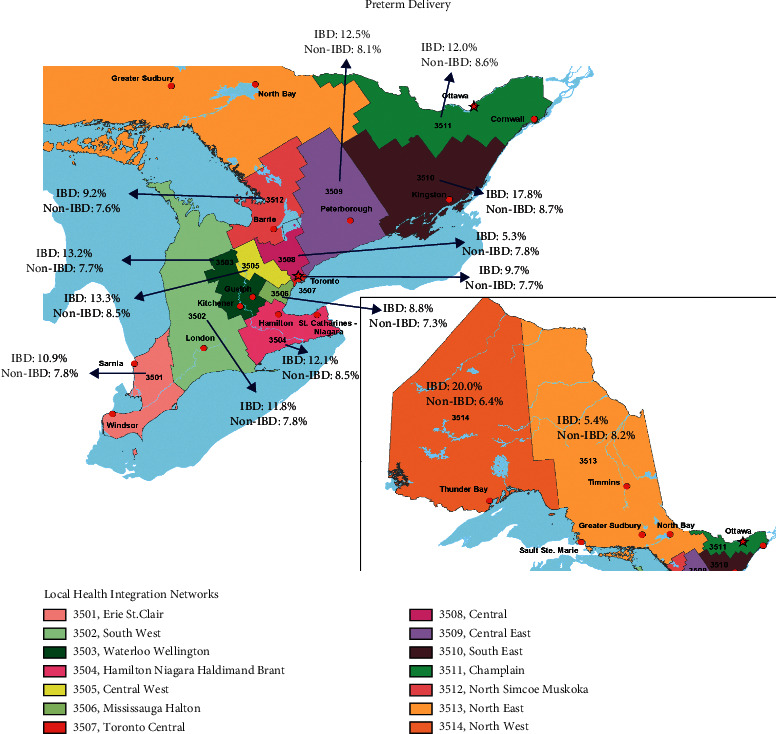
Incidences of preterm delivery in women with and without inflammatory bowel disease (IBD) across the 14 Local Health Integration Networks in Ontario.

The rates of preterm delivery, stratified by each LHIN, in women with and without IBD are summarized in Supplementary Table 1 and Figure 1. There appeared to be geographical variation in the odds of preterm delivery across Ontario, with the highest odds of preterm delivery in the North West LHIN (aOR 2.78, 95% CI, 1.03–7.46), South East LHIN (aOR 2.13, 95% CI, 1.13–4.02), Waterloo Wellington LHIN (aOR 2.04, 95% CI, 1.21–3.45), South West LHIN (aOR 1.84, 95% CI, 1.16–2.91), Central LHIN (aOR 1.71, 95% CI, 1.12–2.59), and Hamilton Niagara Haldimand Brant LHIN (aOR 1.52, 95% CI, 1.04–2.23) (Table 3). There were no differences in preterm delivery in women with and without IBD in the other regions of Ontario.

### 3.2. Rates of Small for Gestational Age Stratified by LHIN

**Figure 2:**
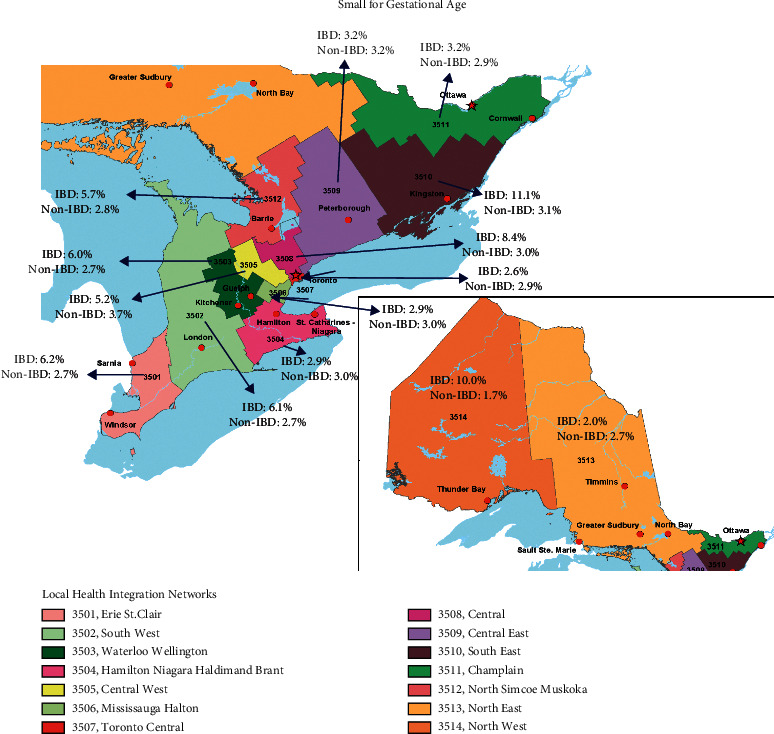
Incidences of infants small for gestational age in women with and without inflammatory bowel disease (IBD) across the 14 Local Health Integration Networks in Ontario.

The rates of SGA, stratified by LHIN, in women with and without IBD are summarized in Supplementary Table 1 and Figure 2. Similar to preterm birth, women with IBD living in the North West LHIN also appeared to have the highest odds of delivering an infant with SGA (aOR 5.66, 95% CI, 1.67–19.14) (Table 4). Otherwise, there remained geographical variation in the odds of infants born SGA to women with IBD across the province with the highest rates observed in the South East LHIN (aOR 2.74, 95% CI, 1.18–6.38), South West LHIN (aOR 2.61, 95% CI, 1.44–4.73), North Simcoe Muskoka LHIN (aOR 2.53, 95% CI, 1.01–6.34), Erie St. Clair LHIN (aOR 2.33, 95% CI, 1.07–5.06), Waterloo Wellington LHIN (aOR 2.22, 95% CI, 1.03–4.81), and Central LHIN (aOR 1.89, 95% CI, 1.05–3.40).

### 3.3. Rates of Cesarean Delivery Stratified by LHIN

Women with IBD had higher rates of Cesarean delivery (917/2466, 37.2%) compared to those without IBD (417,543/1,280,493, 32.6%) (*p* < 0.005) (Table 1 and Supplementary Table 2). The aOR for C-section in women with IBD compared to those without IBD was 1.34 (95% CI, 1.22–1.49) and 1.19 (95% CI, 1.03–1.37) for UC, and 1.53 (95% CI, 1.33–1.75) for CD (Table 5).

There appeared to be significant geographical variation in Cesarean delivery rates in women with IBD across all 14 LHINs. Similar to preterm delivery and SGA, the odds of Cesarean delivery in women with IBD were highest in the North West LHIN (aOR 2.48, 95% CI, 1.11–5.55). It also remained significantly higher in the Hamilton Niagara Haldimand Brant LHIN, Toronto Central LHIN, Central LHIN, and Champlain LHIN.

For women with UC, the odds of Cesarean delivery were only increased compared to those without IBD in the South West LHIN (aOR 2.04, 95% CI, 1.26–3.31), whereas there was no difference between the two groups in the other 13 LHINs (Table 5). For women with CD, however, the odds of Cesarean delivery were increased in the Hamilton Niagara Haldimand Brant LHIN (1.54, 95% CI, 1.05–2.27), Central West LHIN (2.14, 95% CI, 1.12–3.84), Central LHIN (1.65, 95% CI, 1.07–2.55), Champlain LHIN (1.66, 95% CI, 1.08–2.53), and North Simcoe Muskoka LHIN (2.45, 95% CI, 1.30–4.61).

## 4. Discussion

Previous studies have demonstrated significant geographical variation in the care of patients with IBD, with a higher risk of hospitalizations and need for intestinal surgery in those living in low population density regions [11]. Similarly, women living in these regions may also be at a higher risk of maternal and perinatal morbidity due to lack of access to specialist obstetrical care [12]. Whether this applies to women with IBD during pregnancy is important to determine, given the inherently increased risk of adverse obstetrical outcomes such as preterm birth [17] and infants born SGA [4].

Indeed, we demonstrate significant regional variation in the rates of adverse obstetrical outcomes in women with IBD in this large population-based study. In particular, there was more than a 2-fold increase in preterm delivery and a 5-fold increase in rates of infants born with SGA in women with IBD, compared to those without IBD, in the northernmost regions of Ontario. Furthermore, there were no significant differences observed in these outcomes in women with and without IBD in large urban centers such as Toronto and the surrounding areas.

Interestingly, the highest rates of preterm delivery and infants born with SGA in our study were found in areas with the fewest practicing gastroenterologists per 100,000 capita and where patients receive the lowest continuous care for their underlying IBD [18]. It has been well established that a greater distance to an IBD referral center is associated with an increased risk of adverse outcomes such as intestinal surgery [19] and that these poor outcomes can be improved by increased access to an IBD specialist [20]. Furthermore, early access to a dedicated preconception and IBD clinic can reduce the relapse of IBD during pregnancy [21], and even a single preconception consultation with a gastroenterologist can improve patient knowledge and promote medication adherence during pregnancy [22]. This may reduce the risk of adverse pregnancy outcomes such as infants born with low birth weight [21]. Whether the increased rates of adverse pregnancy outcomes in our study in northern Ontario were due to a lack of access to specialised gastroenterology and IBD care and subsequent disease activity during pregnancy remains to be determined in future studies.

Similarly, a lack of access to dedicated obstetrical care and inadequate prenatal visits may also have contributed to the higher rates of adverse outcomes in more remote regions of Ontario. Inadequate prenatal care has been associated with an increased likelihood of preterm delivery infants born SGA and even perinatal mortality [23]. Furthermore, compared to those living in urban centers, women living in rural areas were likely to have a lower number of prenatal visits and reduced access to obstetricians, which in turn increased the risk of preterm birth [12]. The farther the distance the patient lived from the obstetrical hospital, the higher the probability of preterm birth [24].

Interestingly, our finding that the highest likelihood of SGA in more remote areas is not consistent with what has been described previously in women without IBD [12]. In fact, women without IBD in more rural areas were more likely to deliver babies large for gestational age rather than SGA, compared to those in more urban centers [12]. This is possibly due to higher rates of maternal obesity and diabetes in these more remote areas resulting in larger fetal weights [25]. This higher risk of SGA in women with IBD may then rather be explained by other disease-specific factors such as underlying inflammation [4], older age at conception [26], and dietary indiscretions [27], all of which are also inherent risk factors for SGA [28] though this remains to be confirmed in future research.

Finally, we also demonstrated significant regional variation of rates in Cesarean delivery across the province, with more significant differences observed in women with CD, whereas there was minimal variation in rates of Cesarean delivery observed across the province in those without IBD (26.8% to 33.1%), the rates of Cesarean delivery varied quite dramatically, particularly in those with CD, where anywhere from 25% to 54.5% of women underwent this mode of delivery depending on geographical location. The minimal variation in Cesarean delivery rates in all women (regardless of IBD diagnosis) across all LHINs was previously established [29], suggesting that the variation we observed in Cesarean delivery in women with IBD is likely due to disease-related factors. Current indications for Cesarean delivery in this population include active perianal disease and possible prior ileal-anal anastomosis [7], though a majority of patients may undergo Cesarean delivery for other reasons such as lack of fetal descent or breech presentation [8]. It remains to be determined then whether the regional variation we observed in Cesarean delivery rates in women with IBD is due to lack of knowledge on indications for Cesarean delivery in this population or specific patient-related factors.

To our knowledge, this is the first study to describe regional variations in adverse pregnancy outcomes in women with IBD. The inclusion of a large number of pregnancies in women with and without IBD allowed for accurate estimates of these outcomes. The population-based design allows for the generalizability of the rates of adverse pregnancy outcomes in women with UC and CD. Ontario is Canada's most populous province, and though all residents have universal access to healthcare, not all have easy access to specialty care due to the low population density [13]. As such, our findings are likely applicable to other jurisdictions where rural and urban disparities exist in access to care. In fact, it is likely that this study underestimates the potential regional disparity in adverse outcomes in different healthcare systems where universal healthcare is not available. Finally, the algorithms used to identify patients with IBD have been previously validated and have high accuracy minimizing misclassification bias [15].

However, there remain inherent limitations. Firstly, our analysis only focused on two adverse pregnancy outcomes: preterm birth and infants born with SGA. However, compared to other adverse pregnancy outcomes, these two have been consistently shown to occur at higher rates in women with IBD than those without [30]. Secondly, the administrative database used to identify pregnancies only captures events occurring within a hospital setting and not those occurring in outpatient clinics or at home. Nonetheless, it includes more than 98% of all births occurring in Ontario [16], and there is no reason to believe that rates of out-of-hospital deliveries differ between women with and without IBD. Furthermore, our use of administrative data prevents any further description of IBD phenotype and severity, particularly as disease activity has been associated with poor pregnancy outcomes [3]. Similarly, potential factors that may also increase adverse pregnancy outcomes such as smoking, maternal weight, and socioeconomic status [31] were not available in the datasets and as such could not be controlled for in our analysis. Given the lack of validated algorithms, we were also unable to adjust for certain comorbidities, such as celiac disease [32], that may also increase the risk of adverse pregnancy outcomes. Future studies should explore this potential confounder, particularly as a celiac disease may be associated with IBD [33]. Finally, due to lack of patient-level data, we were unable to determine factors associated with the regional variations in Cesarean delivery rates in women with IBD and the individual reasons for this mode of delivery (i.e., elective vs. emergency Cesarean delivery).

## 5. Conclusions

Overall, the odds of preterm delivery and infants born SGA, as well as Cesarean delivery, appear to be higher in women with IBD, but there remains significant geographical variation, with the highest rates of these outcomes observed in more remote locations. Whether these observations are due to inherent disease-related factors or access to gastroenterology or prenatal care remains to be determined. Nonetheless, it remains imperative to determine differences in care amongst women with IBD during pregnancy to ensure that care during pregnancy is standardized and every individual receives optimal evidence-based treatment during this otherwise high-risk period.

## Figures and Tables

**Table 1:** Baseline characteristics of included patients and number of patients stratified by Local Health Integration Networks (LHINs).

	Inflammatory bowel disease (*n* = 2466 births)	Noninflammatory bowel disease (*n* = 1,280,493 births)	Total (*n* = 1,282,959 mothers)
*Age at conception (mean* ± *SD)*	28.9 ± 4.5	28.8 ± 5.4	28.8 ± 5.4

*Mean neighbourhood Income quintile (n, %)*			
Q1 (lowest)	369 (15.0%)	294,035 (23.0%)	294,404 (22.9%)
Q2	459 (18.6%)	262,666 (20.5%)	263,125 (20.5%)
Q3	538 (21.8%)	258,098 (20.2%)	258,636 (20.2%)
Q4	632 (25.6%)	256,696 (20.0%)	257,328 (20.1%)
Q5 (highest)	462 (18.7%)	202,489 (15.8%)	202,951 (15.8%)

*Number of patients included per LHIN (n, %)*			
Erie St. Clair (LHIN 1)	129 (5.2%)	61,968 (4.8%)	62,097 (4.8%)
South West (LHIN 2)	212 (8.6%)	87,879 (6.9%)	88,091 (6.9%)
Waterloo Wellington (LHIN 3)	151 (6.1%)	74,249 (5.8%)	74,400 (5.8%)
Hamilton Niagara Haldimand Brant (LHIN 4)	314 (12.7%)	124,774 (9.7%)	125,099 (9.7%)
Central West (LHIN 5)	135 (5.5%)	101,142 (7.9%)	101,277 (7.9%)
Mississauga Halton (LHIN 6)	204 (8.3%)	114,474 (8.9%)	114,678 (8.9%)
Toronto Central 7 (LHIN 7)	195 (7.9%)	133,558 (10.4%)	133,753 (10.4%)
Central (LHIN 8)	262 (10.6%)	170,466 (13.3%)	170,728 (13.3%)
Central East (LHIN 9)	248 (10.1%)	147,513 (11.5%)	147,761 (11.5%)
South East (LHIN 10)	90 (3.6%)	39,429 (3.1%)	39,519 (3.1%)
Champlain (LHIN 11)	250 (10.1%)	118,216 (9.2%)	118,466 (9.2%)
North Simcoe Muskoka (LHIN 12)	87 (3.5%)	37,759 (2.9%)	37,846 (2.9%)
North East (LHIN 13)	149 (6.0%)	46,045 (3.6%)	46,194 (3.6%)
North West (LHIN 14)	40 (1.6%)	21,580 (1.7%)	21,620 (1.7%)

**Table 2:** Rates of preterm delivery, infants born small for gestational age, and Cesarean delivery for women with and without inflammatory bowel disease.

	Inflammatory bowel disease (*n* = 2466 births)	Noninflammatory bowel disease (*n* = 1,280,493 births)	*p*	Age-adjusted odds ratio (95% CI)
Preterm delivery (*n*, %)	293 (11.9%)	102,274 (8.0%)	<0.001	1.56 (1.35–1.79)
Small for gestational age (*n*, %)	117 (4.7%)	38,261 (3.0%)	<0.001	1.52 (1.23, 1.88)
Cesarean delivery (*n*, %)	917 (37.2%)	417,543 (32.6%)	<0.001	1.34 (1.22–1.49)

**Table 3:** Age-adjusted odds ratios for preterm birth stratified by Local Health Integration Networks.

	Age-adjusted odds ratio for preterm birth (aOR, 95% CI)	*p*
Erie St. Clair (LHIN 1)	1.60 (0.89, 2.89)	0.12
South West (LHIN 2)	1.84 (1.16, 2.91)	**0.009**
Waterloo Wellington (LHIN 3)	2.04 (1.21, 3.45)	**0.008**
Hamilton Niagara Haldimand Brant (LHIN 4)	1.52 (1.04, 2.23)	**0.03**
Central West (LHIN 5)	1.60 (0.90, 2.83)	0.11
Mississauga Halton (LHIN 6)	1.42 (0.84, 2.39)	0.19
Toronto Central 7 (LHIN 7)	1.22 (0.70, 2.13)	0.48
Central (LHIN 8)	1.71 (1.12, 2.59)	**0.01**
Central East (LHIN 9)	1.45 (0.93, 2.28)	0.10
South East (LHIN 10)	2.13 (1.13, 4.02)	**0.02**
Champlain (LHIN 11)	1.62 (1.06, 2.46)	**0.02**
North Simcoe Muskoka (LHIN 12)	1.50 (0.71, 3.16)	0.29
North East (LHIN 13)	0.53 (0.23, 1.22)	0.14
North West (LHIN 14)	2.78 (1.03, 7.46)	**0.04**

Bold *p* values demonstrate statistical significance.

**Table 4:** Age-adjusted odds ratios for small for gestational age stratified by Local Health Integration Networks.

	Age-adjusted odds ratio for small for gestational age (aOR, 95% CI)	*p*
Erie St. Clair (LHIN 1)	2.33 (1.07, 5.06)	**0.03**
South West (LHIN 2)	2.61 (1.44, 4.73)	**0.002**
Waterloo Wellington (LHIN 3)	2.22 (1.03, 4.81)	**0.04**
Hamilton Niagara Haldimand Brant (LHIN 4)	1.03 (0.51, 2.10)	0.93
Central West (LHIN 5)	1.23 (0.50, 3.02)	0.66
Mississauga Halton (LHIN 6)	1.02 (0.42, 2.50)	0.96
Toronto Central 7 (LHIN 7)	0.85 (0.31, 2.30)	0.75
Central (LHIN 8)	1.89 (1.05, 3.40)	**0.03**
Central East (LHIN 9)	0.95 (0.42, 2.15)	0.91
South East (LHIN 10)	2.74 (1.18, 6.38)	**0.02**
Champlain (LHIN 11)	1.18 (0.55, 2.52)	0.67
North Simcoe Muskoka (LHIN 12)	2.53 (1.01, 6.34)	**0.04**
North East (LHIN 13)	0.82 (0.26, 2.59)	0.73
North West (LHIN 14)	5.66 (1.67, 19.14)	**0.005**

Bold *p* values demonstrate statistical significance.

**Table 5:** Age-adjusted odds ratio for Cesarean delivery for women with inflammatory bowel disease, stratified by ulcerative colitis and Crohn's disease.

	Age-adjusted odds ratio for cesarean delivery		
	Inflammatory bowel disease (aOR, 95% CI)	Ulcerative colitis (aOR, 95% CI)	Crohn's disease (aOR, 95% CI)
All	1.34 (1.22–1.49)	1.19 (1.03–1.37)	1.53 (1.33–1.75)
Erie St. Clair (LHIN 1)	1.18 (0.76–1.81)	0.94 (0.50–1.75)	1.47 (0.81–2.68)
South West (LHIN 2)	1.42 (0.99–2.02)	2.04 (1.26–3.31)	0.96 (0.56–1.64)
Waterloo Wellington (LHIN 3)	1.04 (0.67–1.59)	0.81 (0.43–1.52)	1.31 (0.73–2.37)
Hamilton Niagara Haldimand Brant (LHIN 4)	1.44 (1.09–1.91)	1.34 (0.89–2.02)	1.54 (1.05–2.27)
Central West (LHIN 5)	1.27 (0.84–1.93)	0.73 (0.38–1.38)	2.14 (1.12–3.84)
Mississauga Halton (LHIN 6)	1.27 (0.89–1.82)	1.06 (0.64–1.75)	1.57 (0.94–2.63)
Toronto Central 7 (LHIN 7)	1.51 (1.06–2.13)	1.56 (0.97–2.53)	1.44 (0.87–2.40)
Central (LHIN 8)	1.46 (1.08–1.96)	1.31 (0.87–1.97)	1.65 (1.07–2.55)
Central East (LHIN 9)	1.15 (0.84–1.59)	1.02 (0.65–1.61)	1.30 (0.83–2.04)
South East (LHIN 10)	1.03 (0.60–1.75)	1.21 (0.44–3.32)	0.97 (0.52–1.81)
Champlain (LHIN 11)	1.44 (1.05–1.96)	1.23 (0.78–1.94)	1.66 (1.08–2.53)
North Simcoe Muskoka (LHIN 12)	1.63 (0.99–2.67)	0.81 (0.34–1.94)	2.45 (1.30–4.61)
North East (LHIN 13)	1.31 (0.89–1.93)	0.99 (0.57–1.76)	1.70 (0.99–2.88)
North West (LHIN 14)	2.48 (1.11–5.55)	2.09 (0.69–6.29)	3.02 (0.92–9.92)

## Data Availability

The dataset from this study is held securely in coded form at ICES. However, legal data sharing agreements between ICES and data providers (e.g., healthcare organizations and government) prohibit ICES from making the dataset publicly available; access may be granted to those who meet prespecified criteria for confidential access, available at www.ices.on.ca/DAS (email: das@ices.on.ca). The full dataset creation plan and underlying analytic code are available from the authors upon request, understanding that the computer programs may rely upon coding templates or macros that are unique to ICES, and therefore, they are either inaccessible or may require modification.

## Supplementary Materials

Supplementary Materials Supplementary Table 1: rates of preterm delivery and small for gestational age in women with and without inflammatory bowel disease stratified by Ontario's 14 Local Health Integration Networks. Supplementary Table 2: cesarean delivery rates in women with and without inflammatory bowel disease stratified by Ontario's 14 Local Health Integration Networks.
