# Supplementary material for: Regional Variation in Pregnancy Outcomes amongst Women in Inflammatory Bowel Disease: A Population-Based Cohort Study
Source: Can J Gastroenterol Hepatol. 2021 Nov 29;2021:3037128. doi: 10.1155/2021/3037128 (PMC8648466; doi:10.1155/2021/3037128)
Supplement: Supplementary Materials — Supplementary Table 1: rates of preterm delivery and small for gestational age in women with and without inflammatory bowel disease stratified by Ontario's 14 Local Health Integration Networks. Supplementary Table 2: cesarean delivery rates in women with and without inflammatory bowel disease stratified by Ontario's 14 Local Health Integration Networks. [file 3037128.f1.docx]

|  | **Inflammatory Bowel Disease** | | | **Non-Inflammatory Bowel Disease** | | |
| --- | --- | --- | --- | --- | --- | --- |
|  | **Total Births**  **(n, %)** | **Preterm Delivery**  **(n, %)** | **Small for Gestational Age**  **(n, %)** | **Total**  **Births**  **(n, %)** | **Preterm Delivery**  **(n, %)** | **Small for Gestational Age**  **(n, %)** |
| Erie St. Clair (LHIN 1) | 129 | 14 (10.9%) | 8 (6.2%) | 61, 968 | 4807 (7.8%) | 1,685 (2.7%) |
| South West (LHIN 2) | 212 | 25 (11.8%) | 13 (6.1%) | 87, 879 | 6,853 (7.8%) | 2,487 (2.8%) |
| Waterloo Wellington (LHIN 3) | 151 | 20 (13.2%) | 9 (6.0%) | 74, 249 | 5,741 (7.7%) | 1,998 (2.7%) |
| Hamilton Niagara Haldimand Brant (LHIN 4) | 314 | 38 (12.1%) | 9 (2.9%) | 124, 774 | 10,610 (8.5%) | 3,755 (3.0%) |
| Central West (LHIN 5) | 135 | 18 (13.3%) | 7 (5.2%) | 101, 142 | 8,623 (8.5%) | 3,705 (3.7%) |
| Mississauga Halton (LHIN 6) | 204 | 18 (8.8%) | 6 (2.9%) | 114, 474 | 8,410 (7.3%) | 3,449 (3.0%) |
| Toronto Central 7 (LHIN 7) | 195 | 19 (9.7%) | <5 | 133, 558 | 10,218 (7.7%) | 3,920 (2.9%) |
| Central (LHIN 8) | 262 | 40 (15.3%) | 22 (8.4%) | 170, 466 | 13,231 (7.8%) | 5,197 (3.0%) |
| Central East (LHIN 9) | 248 | 31 (12.5%) | 8 (3.2%) | 147, 513 | 12,015 (8.1%) | 4,696 (3.2%) |
| South East (LHIN 10) | 90 | 16 (17.8%) | 10 (11.1%) | 39, 429 | 3,446 (8.7%) | 1,224 (3.1%) |
| Champlain (LHIN 11) | 250 | 30 (12.0%) | 8 (3.2%) | 118, 216 | 10,139 (8.6%) | 3,431 (2.9%) |
| North Simcoe Muskoka (LHIN 12) | 87 | 8 (9.2%) | <5 | 37, 759 | 2,887 (7.6%) | 1,039 (2.8%) |
| North East (LHIN 13) | 149 | 8 (5.4%) | <5 | 46, 045 | 3,780 (8.2%) | 1,258 (2.7%) |
| North West (LHIN 14) | 40 | 5 (20.0%) | <5 | 21, 580 | 1,382 (6.4%) | 375 (1.7%) |

**Supplementary Table 1:** Rates of preterm delivery and small for gestational age in women with and without inflammatory bowel disease stratified by Ontario’s 14 local health integration networks

|  | **Non-inflammatory Bowel Disease** | **Inflammatory Bowel Disease** | **p**  **(IBD vs. non-IBD)** | **Ulcerative Colitis** | **p**  **(UC vs. non-IBD)** | **Crohn’s disease** | **p**  **(CD vs. non-IBD)** |
| --- | --- | --- | --- | --- | --- | --- | --- |
| Erie St. Clair (LHIN 1) | 14,302/49,320 (29.0%) | 31/96  (32.3%) | 0.477 | 14/49  (28.6%) | 0.94 | 17/47 (36.2%) | 0.28 |
| South West (LHIN 2) | 18,424/68,631 (26.8%) | 47/140  (33.6%) | 0.07 | 29/68  (42.6%) | **0.003** | 18/72 (25.0%) | 0.72 |
| Waterloo Wellington (LHIN 3) | 17,600/59,392 (29.6%) | 30/100  (30.0%) | 0.94 | 13/51  (25.5%) | 0.52 | 17/49 (34.7%) | 0.44 |
| Hamilton Niagara Haldimand Brant (LHIN 4) | 30,971/101,604 (30.5%) | 82/209  (39.2%) | **0.006** | 38/100 (38.0%) | 0.10 | 44/109 (40.4%) | **0.03** |
| Central West (LHIN 5) | 26,480/82,398 (32.1%) | 36/95  (37.9%) | 0.23 | 13/49  (26.5%) | 0.40 | 23/46 (50.0%) | **0.009** |
| Mississauga Halton (LHIN 6) | 26,502/94,928 (27.9%) | 45/139  (32.4%) | 0.24 | 22/76  (28.9%) | 0.84 | 23/63 (36.5%) | 0.13 |
| Toronto Central 7 (LHIN 7) | 34,184/110,581 (30.9%) | 54/134  (40.3%) | **0.02** | 29/70  (41.4%) | 0.06 | 25/64 (39.1%) | 0.16 |
| Central (LHIN 8) | 43,270/140,531 (30.8%) | 73/187  (39.0%) | **0.01** | 37/101 (36.6%) | 0.21 | 36/86 (41.9%) | **0.03** |
| Central East (LHIN 9) | 39,784/123,936 (32.1%) | 58/172  (33.7%) | 0.65 | 28/87  (32.2%) | 0.99 | 30/85 (35.3%) | 0.53 |
| South East (LHIN 10) | 10,104/32,679 (30.9%) | 20/64  (31.3%) | 0.95 | 6/17  (35.3%) | 0.70 | 14/47 (29.8%) | 0.87 |
| Champlain (LHIN 11) | 29,471/97,016 (30.4%) | 65/172  (37.8%) | **0.03** | 29/82  (35.4%) | 0.33 | 36/90 (40.0%) | 0.05 |
| North Simcoe Muskoka (LHIN 12) | 10,233/30,907 (33.1%) | 28/64  (43.8%) | 0.07 | 7/25  (28.0%) | 0.59 | 21/39 (53.8%) | **0.005** |
| North East (LHIN 13) | 12,728/39,225 (32.4%) | 43/111  (38.7%) | 0.16 | 17/55  (32.7%) | 0.96 | 25/56 (44.6%) | 0.05 |
| North West (LHIN 14) | 5,088/17,689  (28.8%) | 12/24  (50.0%) | **0.02** | 6/13  (46.2%) | 0.166 | 6/11 (54.5%) | 0.06 |

**Supplementary Table 2:** Rates of Cesarean delivery in women with and without inflammatory bowel disease stratified by Ontario’s 14 local health integration networks
